# Identification of Mollusc Remains (Bivalve and Gastropod) from Archaeological Sites in Semporna, Sabah

**DOI:** 10.21315/tlsr2022.33.2.10

**Published:** 2022-06-30

**Authors:** Deejay Daxter A. Albert, Velat Bujeng, Stephen Chia

**Affiliations:** Centre for Global Archaeological Research, Universiti Sains Malaysia, 11800 Pulau Pinang, Malaysia

**Keywords:** Mollusc Remains, Archaeomalacology, Archaeological Sites, Semporna, Prehistory, Tinggalan Moluska, Arkeomalakologi, Tapak Arkeologi, Semporna, Prasejarah

## Abstract

This paper discusses the identification of mollusc (bivalve and gastropod) remains from three archaeological sites in Semporna, Sabah, namely Bukit Tengkorak, Melanta Tutup and Bukit Kamiri, dated to the prehistoric period, from 3,000 to 800 years ago. Samples of mollusc remains used in this study were obtained from a series of archaeological excavations conducted at these three sites by the Centre for Global Archaeological Research, Universiti Sains Malaysia (CGAR, USM), Penang in collaboration with the Sabah Museum Department (SMD) from 1994 to 2007. In total, 90 taxa of molluscan species, of which 30 are bivalves and 60 are gastropods, had been identified. Out of 90, there were 55 taxa identified to the species level, of which 18 are bivalves and 37 are gastropods. They consisted mainly of marine species with small numbers of freshwater, brackish and terrestrial species. This study had provided new data and insights into the distribution and exploitation of molluscs by ancient human societies in different environments in Semporna, which will be useful not only for malacological research in the tropics but also for future biological and environmental studies in Sabah, Malaysia as well as for the Southeast Asian and Pacific regions.

## Abstract (ms)

Artikel ini membincangkan tentang identifikasi tinggalan moluska (dwicangkerang dan gastropod) daripada tiga tapak arkeologi di Semporna Sabah, iaitu Bukit Tengkorak, Melanta Tutup dan Bukit Kamiri, daripada zaman prasejarah 3,000 ke 800 tahun yang lalu. Sampel tinggalan moluska yang digunakan dalam kajian ini diambil daripada beberapa siri ekskavasi arkeologi yang dijalankan di tiga tapak oleh Pusat Penyelidikan Arkeologi Global, Universiti Sains Malaysia, Pulau Pinang, dengan kerjasama Jabatan Muzium Sabah daripada tahun 1994 sehingga 2007. Sebanyak 90 taxa spesies moluska telah dikenalpasti iaitu 30 daripadanya adalah dwicangkerang dan 60 adalah gastropod. Daripada 90 taxa tersebut, 55 taxa dikenalpasti pada tahap spesies, manakala 18 adalah dwicangkerang dan 37 adalah gastropod. Kebanyakan taxa tersebut terdiri daripada spesies marin termasuk juga sedikit bilangan daripada spesies air tawar, spesies air payau dan spesies daratan. Kajian ini telah memberikan data dan pandangan baru mengenai taburan dan eksploitasi moluska oleh masyarakat manusia purba dalam persekitaran yang berbeza di Semporna, yang akan berguna bukan sahaja untuk penyelidikan malakologi di kawasan tropika tetapi juga penting untuk kajian biologi dan alam sekitar di Sabah, Malaysia termasuk untuk rantau Asia Tenggara serta Pasifik pada masa depan.

**Highlights**

- Mollusc remains acquired through excavation of three archaeological sites in Semporna, Sabah namely Bukit Tengkorak, Melanta Tutup and Bukit Kamiri were identified.
- 90 taxa of molluscan species had been identified, where 30 are bivalves and 60 are gastropods.
- Species richness, indicated by NTAXA value, between the three sites preliminarily indicates different level of selectivity in gathering molluscs among ancient communities in different sites, possibly due to different proximity of sites to the coastline.

## INTRODUCTION

One of the most common biological materials recovered from archaeological sites during excavations is the remains of bivalve and gastropod from the phylum Mollusca. Animals from the phylum Mollusca usually have a calcium carbonate exoskeleton called the ‘shell’ that is hard and durable in nature that could survive well when buried in the soil (Claassen 1998; Thomas 2015a). Hence, more often than not, only molluscs with shells are found during archaeological excavations, particular from the classes Bivalvia and Gastropoda, while the other classes, especially the shell-less ones like Aplacophora and Monoplacophora are seldom found in the archaeological context (Claassen 1998; Bar-Yosef Mayer 2005; 2007; Reitz & Wing 2008; Thomas 2015a). Mollusc remains are defined as the pieces or parts of the shells of molluscs that are buried underground and uncovered during archaeological excavations. In other words, mollusc remains are dead specimens consisting of only the empty shells of the molluscs. Hence, identification of mollusc remains depends solely on the morphology of the shell. Some archaeologists used the term ‘shell remains’, but the term ‘mollusc remains’ is used because certain animals not from the phylum Mollusca also have shells, such as tortoises, turtles and Crustacean (such as crabs, prawn or mantis shrimp).

In archaeology, the study of mollusc remains in the archaeological contexts is defined as archaeomalacology, which is the sub-field of archaeozoology. The most common contribution in archaeomalacological studies is the interpretation of ancient human diet and subsistence by identifying and quantifying the taxa of mollusc remains found in an archaeological site (Zulkifli *et al*. 1992; Szabo 2009; Gani *et al*. 2013; Bujeng 2018). In most cases, a large number of molluscs remains found in an archaeological site that does not occur naturally there would indicate that they were actually gathered and brought into the sites by humans for their sustenance (Thomas, 2015b). Identifying these mollusc remains can help in determining their species and original habitats, which could provide useful data on past environments, movement of humans and their strategies in searching and gathering, as well as the adaptive change of past humans in exploiting molluscs over time (Shackleton & van Andel 1986; Ono *et al*. 2009; Habu *et al*. 2011). In addition, archaeomalacological study can help determine the exploitation and use of molluscs by ancient human in the production of artefacts such as ornaments and tools (Szabo 2010; Pawlik *et al*. 2015; Langley & O’Connor 2016; Insoll 2021).

In biological science, the study of archaeomalacology could be beneficial in determining the distribution of molluscan species over time. Many biological science studies, especially in malacology, had employed archaeological data and records on mollusc species to determine and map the origin, presence or extinction of certain species in a particular region over time (Tebano & Paulay 2000; Giokas *et al*. 2010; Argente 2016; Mayor & Ancog 2016; Neo *et al*. 2017; Thaman *et al*. 2017). The study of modern-day molluscan species, on the other hand, had also benefited and assisted archaeology in identifying ancient mollusc remains from archaeological sites (Meehan 1982; Rouse & Evans 1994; Welter-Schultes 2008). The same can also be said vice versa especially in terms of shell morphometric (Faulkner 2010; Ash *et al*. 2013; Fiori *et al*. 2019). In addition, Thaman *et al*. (2017) had shown how time-depth perspectives on molluscan species with the incorporation of archaeological data could provide a wealth of information on the ecology, evolution and structure of past marine ecosystems as well as the history of human impacts on the environments. In short, archaeomalacology is a significant field of study that is beneficial not only in the study of the past but also for the present.

Bukit Tengkorak was briefly excavated in 1987 by Peter Bellwood with the assistance of SMD (Bellwood 1989). Following this, several archaeological excavations were conducted from 1994–2007 by a research team headed by Stephen Chia from CGAR, USM in collaboration with the SMD team at Bukit Tengkorak, Melanta Tutup and Bukit Kamiri (Chia 2003; 2008; 2009). These detailed excavations by CGAR, USM at the three sites had uncovered a significant amount of archaeological data and materials which had been radiocarbon dated to the Late Prehistoric period from about 3,000 to 800 years ago (Chia 2003; 2008, 2014; 2016). The archaeological materials excavated from these sites consisted of human burials, ornaments, earthenware sherds, lithic artefacts, bone artefacts, animal and fish bones as well as a considerable amount of mollusc remains.

In terms of mollusc remains excavated from the three archaeological sites in Semporna, those from Bukit Tengkorak had been analysed earlier by Bellwood (1989) and Chia (2003; 2014). However, their analyses which included identifications of the taxa of mollusc samples and the quantification of these specimens were done based on weight and number of identified specimens. This method of analysis is often considered to be outdated now in comparison with the recent archaeomalacological method of analysis. Moreover, some of the mollusc species previously identified by these researchers could have been misidentified since the identifications were not done using the latest taxonomic data from the MolluscaBase (2020) database.

Elsewhere in Semporna, mollusc remains excavated from Melanta Tutup and Bukit Kamiri had been preliminarily analysed by Albert *et al*. (2017; 2018), Bujeng and Albert (2016; 2017) and Chia (2008). The mollusc identification from Melanta Tutup and Bukit Kamiri had so far been reported to class level only by Chia (2008), while Albert *et al*. (2017) and Bujeng and Albert (2016; 2017) had published mollusc species identified from mollusc remains excavated only from the upper layers (0 cm–30 cm depth) of these two sites. In addition, the mollusc assemblage from Melanta Tutup identified by Albert *et al*. (2018) had some outdated nomenclature based on the MolluscaBase (2020) database. For example, the giant clams that were previously listed as a family (Tridacnidae) had now been updated to the subfamily Tridacninae under the family Cardiidae.

Given the various issues and problems in the previous analyses of mollusc remains from the sites in Semporna, this present study intends to conduct a more detailed and comprehensive study of all the mollusc remains excavated from Bukit Tengkorak, Bukit Kamiri dan Melanta Tutup in Semporna, Sabah. This is in order to have a larger sample representation and a more holistic picture and understanding of mollusc species, its distributions and relationships with ancient human societies in Semporna, Sabah. This study will provide the latest data of the identification of mollusc remains from these research sites based on current and updated data from the global, reputable database of MolluscaBase (2020).

## MATERIAL AND METHODS

### Sampling of Mollusc Remains

**Figure 1:**
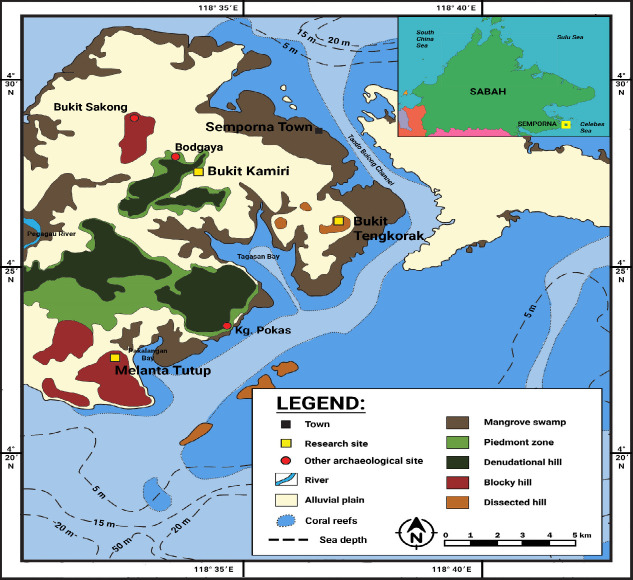
Map of Semporna showing the research sites and the surrounding areas (Source: Adapted and modified from Google Earth; Chia 2008; Kirk 1962; Ho & Kassem 2009).

The mollusc remains were sampled from excavated specimens at the archaeological sites of Bukit Tengkorak (4°27′20.08″N, 118°37′04.3″E), Melanta Tutup (4°21′56.58″N, 118°32′2.04″E) and Bukit Kamiri (4°27′59.70″N, 118°34′8.52″E) in Semporna, Sabah (Fig. 1). Only samples excavated from the projects led by Stephen Chia were used in this study. Samples from Bukit Tengkorak excavated in 1994 to 1995 were stored in the artefact storage of the Archaeology Division of SMD, Kota Kinabalu, and were brought to the archaeology laboratory of SMD to be analysed. Meanwhile, samples from Bukit Tengkorak (excavated in 2002, 20072008), Melanta Tutup (excavated in 2002, 20032006) and Bukit Kamiri (excavated in 2007), which were all stored in the artefact storage of CGAR, were brought to the archaeozoology lab of CGAR to be analysed. All samples, both in SMD and CGAR, were stored in container boxes labelled based on site and year of excavation. Hence, analysis was conducted by transferring box by box from storage to laboratory. This would mean that analysis was done based on site and year of excavation. Samples would then be rearranged based on trench, depth, and then species upon identification.

For example, a box of samples from Bukit Tengkorak excavated in 1994 was transferred to the laboratory. The samples were previously stored in plastic bags labelled according to trench and depth, often disregarding species. As an example, a plastic bag labelled Trench A Spit 1 contained about 30 g of shells. In archaeology, a trench is an excavation grid, while spit is an arbitrarily assigned measurement of depth used as a unit of excavation, such as 10 cm per spit. The plastic bags were then arranged based on trench and spit, and then analysed accordingly. Using the previous example, a plastic bag labelled Trench A Spit 1 would be opened and the samples in it were identified, for example to *Anadara antiquata*, *Gafrarium pectinatum* and *Vasticardium orbita*. Pieces identified to the respective species were then weighed, reference-coded, quantified, photographed and placed separately in different plastic bags. The process was repeated to the next plastic bag (e.g., Trench A Spit 2, Trench B Spit 1 and so on). Once the whole samples in the box were completely analysed, the samples were stored back in the same box, transferred back to the storage and the next box was taken to the laboratory to be analysed.

For Bukit Tengkorak, 8,550 pieces of mollusc remains (83,774.4 g) were analysed, while the samples from Melanta Tutup and Bukit Kamiri consisted of 16,446 pieces (52,290.9 g) and 2,266 pieces (5320.0 g), respectively. All the samples were dry-cleaned using only soft paint brushes, toothbrushes and wooden skewers to remove dirt carefully from the specimens. Wet cleaning was avoided because it may alter or damage the physical structure or colour of the fragile specimens, which could affect the results of our analysis.

### Identification of Species

The methods used in the identification of mollusc species consisted of shell anatomic analysis and taxonomic analysis. Identification of the mollusc specimens was done based on the morphology of the mollusc remains. The identification process of each species was done using the following steps:

1. Each sample was identified to its lowest taxonomic levels by referring to the identification keys provided by Dance (1992), Ridzwan (1993), Poutiers (1998a; 1998b) Abbott and Dance (2000), Abbott (2002), Fiene-Severns *et al*. (2004), Panha and Burch (2004), and Hook (2008).
2. Each sample was also identified as far as possible by comparing the mollusc remains with reference specimens of bivalves and gastropods available at the archaeozoology lab in CGAR, USM.
3. Each species was cross-checked with the database in MolluscaBase (2020) so that all taxa used in this study were the latest and to ensure the consistency as well as accuracy in the identification of the mollusc species (see Albert 2019 for further details).

However, further identification of the samples to the species level required examination of the shell colours, which in this study was very difficult or impossible to do because almost all the samples had lost their original colours after being buried in the soil for thousands of years. In addition, further identification to the species level required examination of the soft flesh and embryonic shells of the mollusc samples which was not possible because all the samples used in this study were dead specimens. Consequently, most of the mollusc samples analysed in this study could only be identified to the lowest taxonomic ranking, which in many cases included the genus, subfamily or family.

### Species Richness

Species richness is an important unit to measure the species diversity for animals in an ecology. Meanwhile, in archaeology, species richness can be used to examine differences in the taxonomic composition of mollusc assemblage from different sites to provide more interpretation on the selectivity or strategies in gathering activities (Nagaoka 2001; Harris *et al*. 2015; 2016). For example, a high value of diversity would indicate low selectivity in the gathering process (Szabo 2009). In this study, species richness was calculated using the NTAXA (number of taxa) method, which is also referred to as taxonomic richness (Lyman 2008; 2015). In an archaeomalacological analysis, NTAXA is best defined as a count of the number of distinct taxa at the highest common taxonomic level so that species richness will not be artificially inflated by taxa that are more easily identified to lower taxonomic levels (Harris *et al*. 2015). As an example, if an assemblage consisted of species identified to *Saccostrea cuccullata* and *Ostreidae* sp., then the NTAXA value would be one (1) rather than two (2), as fragments identified to *Ostreidae* sp. could also possibly be unidentifiable fragments of other species under that family.

## RESULTS AND DISCUSSION

**Figure 2:**
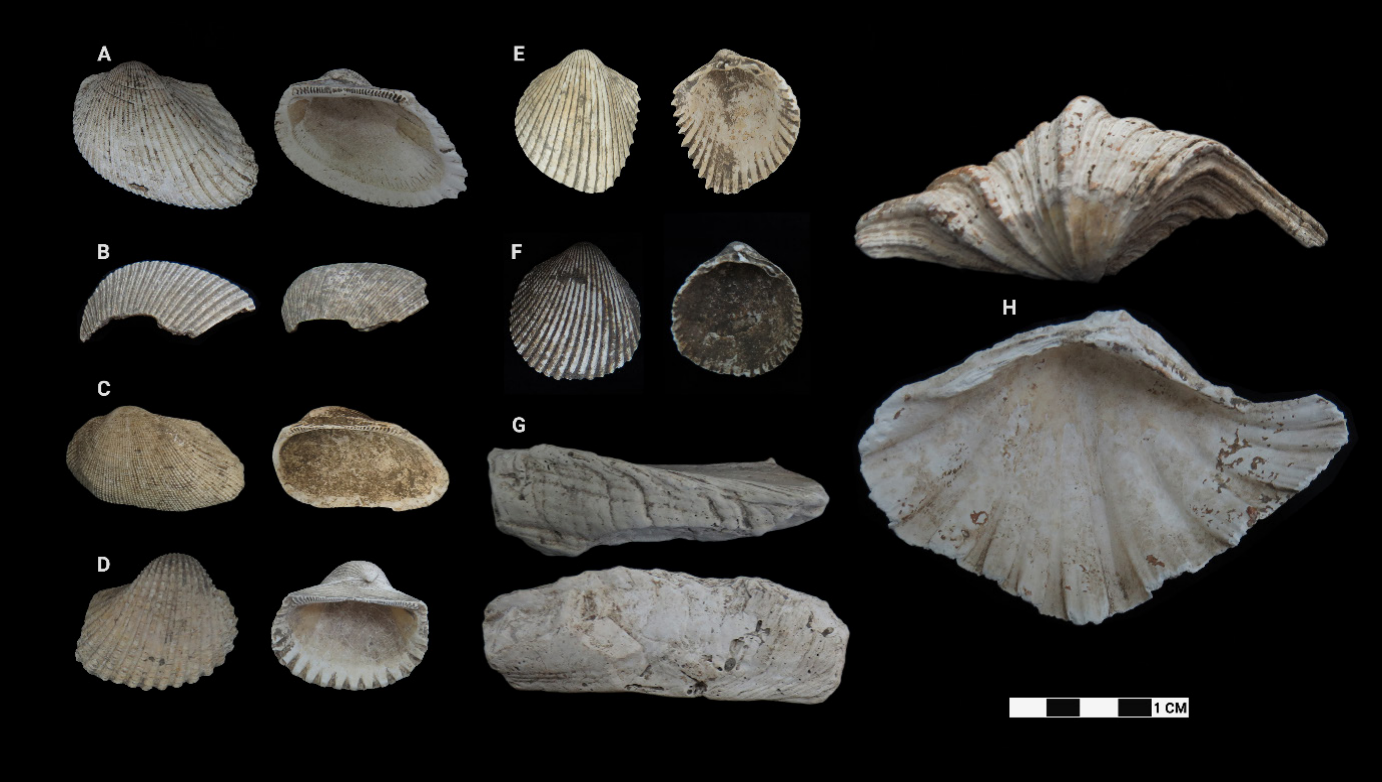
Bivalve specimens identified from archaeological sites in Semporna: (A) *Anadara antiquata* (MT/C/160), (B) *Anadara* sp. (MT/C/104), (C) *Barbatia* sp. (MT/D/Q1/154), (D) *Tegillarca granosa* (MT/C/165), (E) *Fragum unedo* (MT/D/Q1/137), (F) *Vasticardium pectiniforme* (MT/A/141), (G) *Tridacninae* sp. (BT02/P/Q2/29) and (H) *Hippopus hippopus* (MT/D/Q1/209).

**Figure 3:**
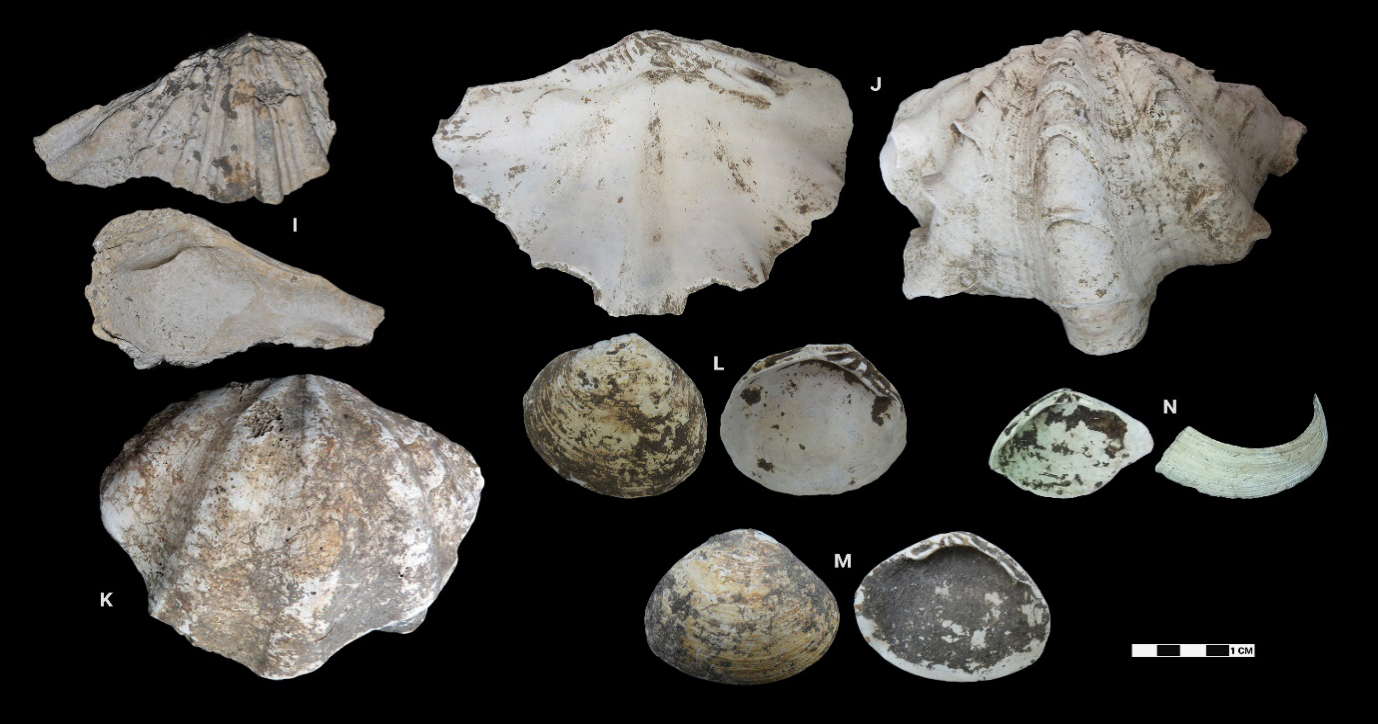
Bivalve specimens identified from archaeological sites in Semporna: (I) *Hippopus* sp. (MT/C/144), (J) *Tridacna squamosa* (MT/D/Q3/81), (K) *Tridacna* sp. (BT/G17/229), (L) *Batissa violacea* (BK/56), (M) *Geloina expansa* (MT/D/Q2/122) and (N) *Geloina* sp. (BK/40).

**Figure 4:**
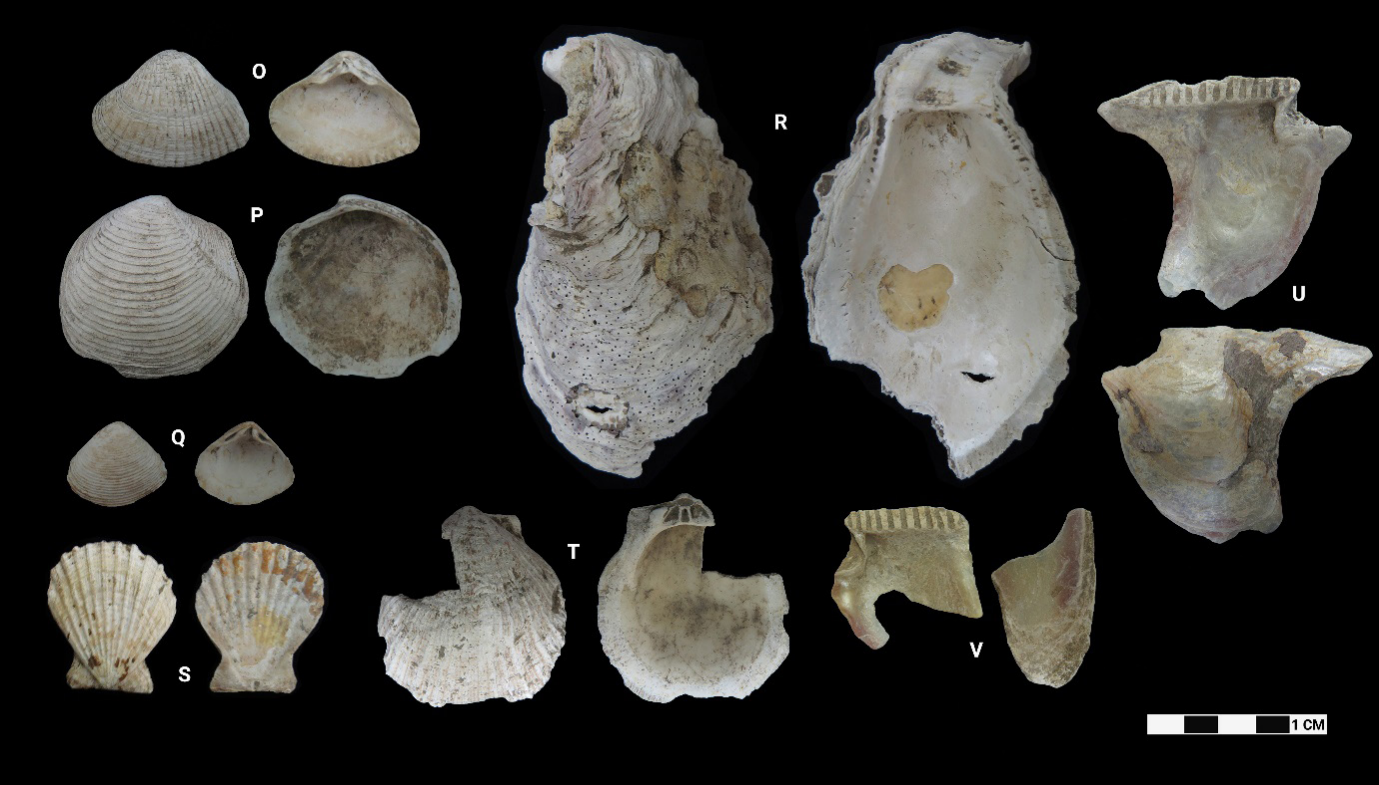
Bivalve specimens identified from archaeological sites in Semporna: (O) *Hemidonax donaciformis* (MT/A/33), (P) *Austriella corrugata* (BT02/P/Q2/23), *Atactodea striata* (MT/B/195), (R) *Saccostrea cucullata* (MT/D/Q1/24), (S) *Decatopecten* sp. (MT/C/280), (T) *Spondylus* sp. (MT/C/98), (U) *Isognomon isognomum* (MT/C/94) and (V) *Isognomon* sp. (MT/C/148).

**Figure 5:**
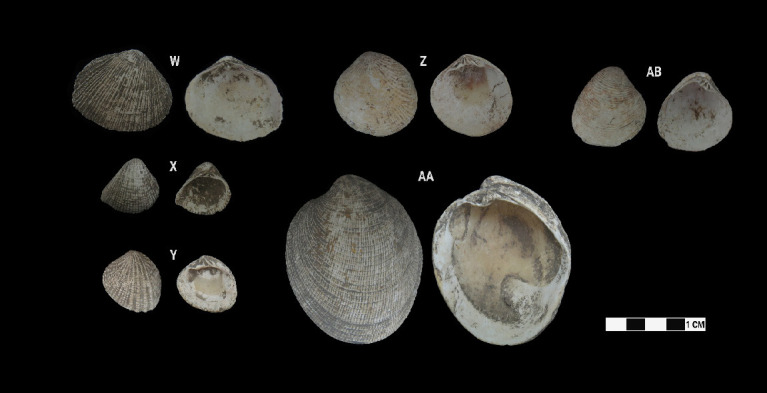
Bivalve specimens identified from archaeological sites in Semporna: (W) *Asaphis violascens* (BT/B/188), (X) *Anomalodiscus squamosus*, (Y) *Gafrarium pectinatum* (MT/C/157), (Z) *Gafrarium divaricatum* (MT/C/93), (AA) *Periglypta puerpera* (MT/D/Q1/184) and (AB) *Pelecyora* sp. (MT/B/194).

**Figure 6:**
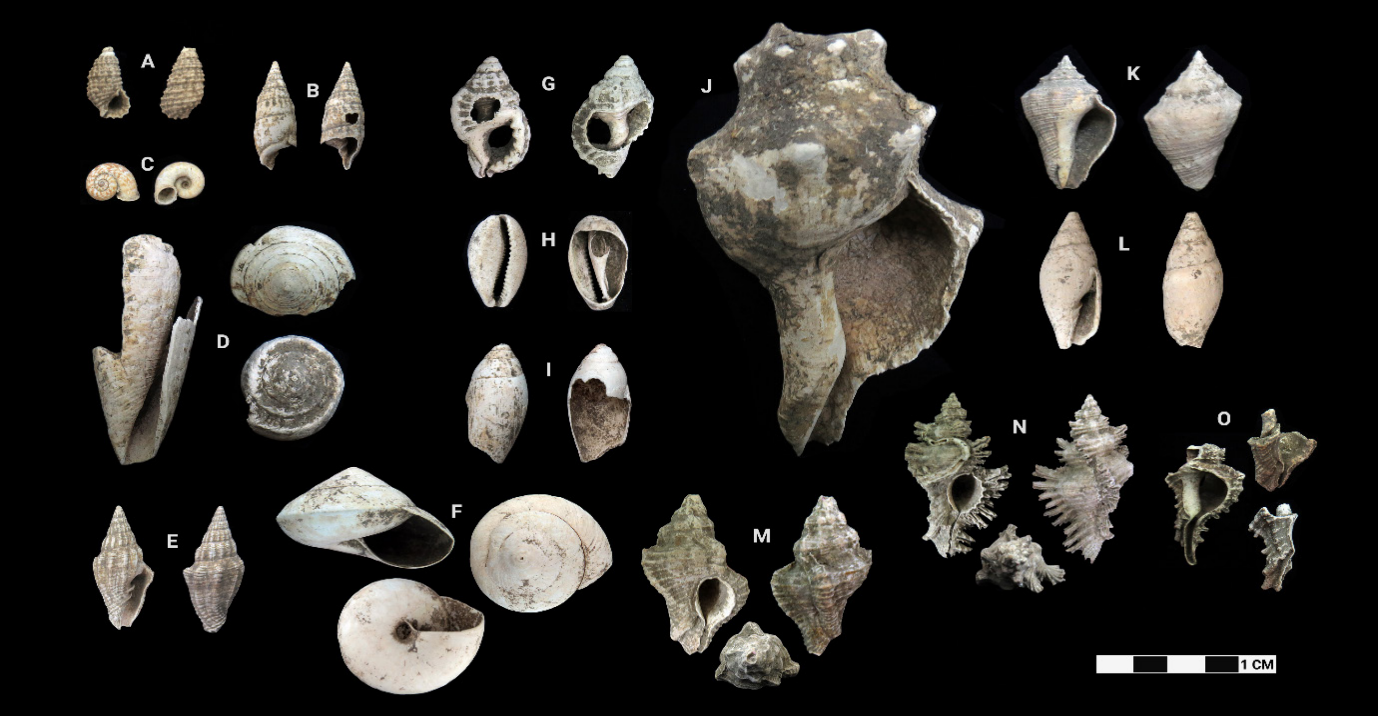
Gastropod specimens identified from archaeological sites in Semporna: (A) *Clypeomorus batillariaeformis* (MT/A/122), (B) *Rhinoclavis vertagus* (MT/C/121), (C) *Opisthoporus* sp. (MT/A/22) (D) *Conus* sp. (MT/D/Q3/115), (E) *Vexillum rugosum* (MT/D/Q2/44), (F) *Cyclophorus* sp. (MT/D/Q3/158), (G) *Monoplex* sp. (MT/D/Q2/161), (H) *Cypraea* sp. (MT/D/Q1/9), (I) *Ellobium* sp. (MT/D/Q4/135), (J) *Pleuroploca* sp. (BT/G17/177), (K) *Volema myristica* (MT/C/127), (L) *Nebularia eremitarum* (MT/D/Q4/71), (M) *Chicoreus capucinus* (MT/C/72), (N) *Chicoreus brunneus* (MT/C/71) and (O) *Chicoreus* sp. (MT/C/73).

**Figure 7:**
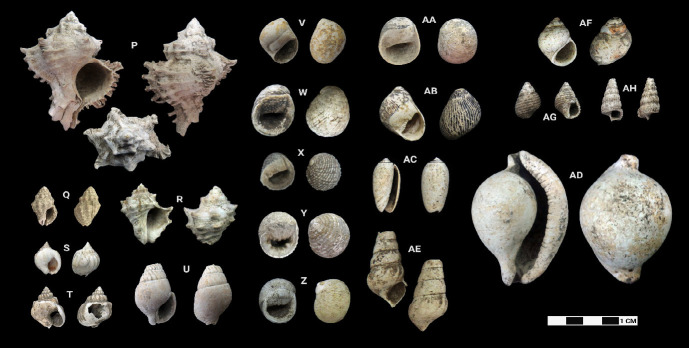
Gastropod specimens identified from archaeological sites in Semporna: (P) *Hexaplex cichoreum* (MT/C/74), (Q) *Drupella margariticola* (MT/D/Q1/124), (R) *Reishia bitubercularis* (MT/D/Q1/126), (S) *Nassarius arcularia* (MT/B/202), (T) *Nassarius coronatus* (MT/D/Q2/137), (U) *Nassarius dorsatus* (MT/A/119), (V) *Polinices* sp. (MT/D/Q2/62), (W) *Nerita albicilla* (MT/C/24), (X) *Nerita histrio* (MT/C/84), (Y) *Nerita plicata* (MT/C/133), (Z) *Nerita undata* (MT/C/139), (AA) *Neritina* cf. *pulligera* (MT/C/23), (AB) *Vittina turrita* (MT/C/81), (AC) *Oliva* sp. (MT/D/Q2/42), (AD) *Ovula ovum* (BT07/A/3), (AE) *Sulcospira* spp. (MT/D/Q2/65), (AF) *Paludomus everetti* (BK/44), (AG) *Planaxis sulcatus* (MT/B/166) and (AH) *Cerithidea* cf. *quoyii* (MT/B/168).

**Figure 8:**
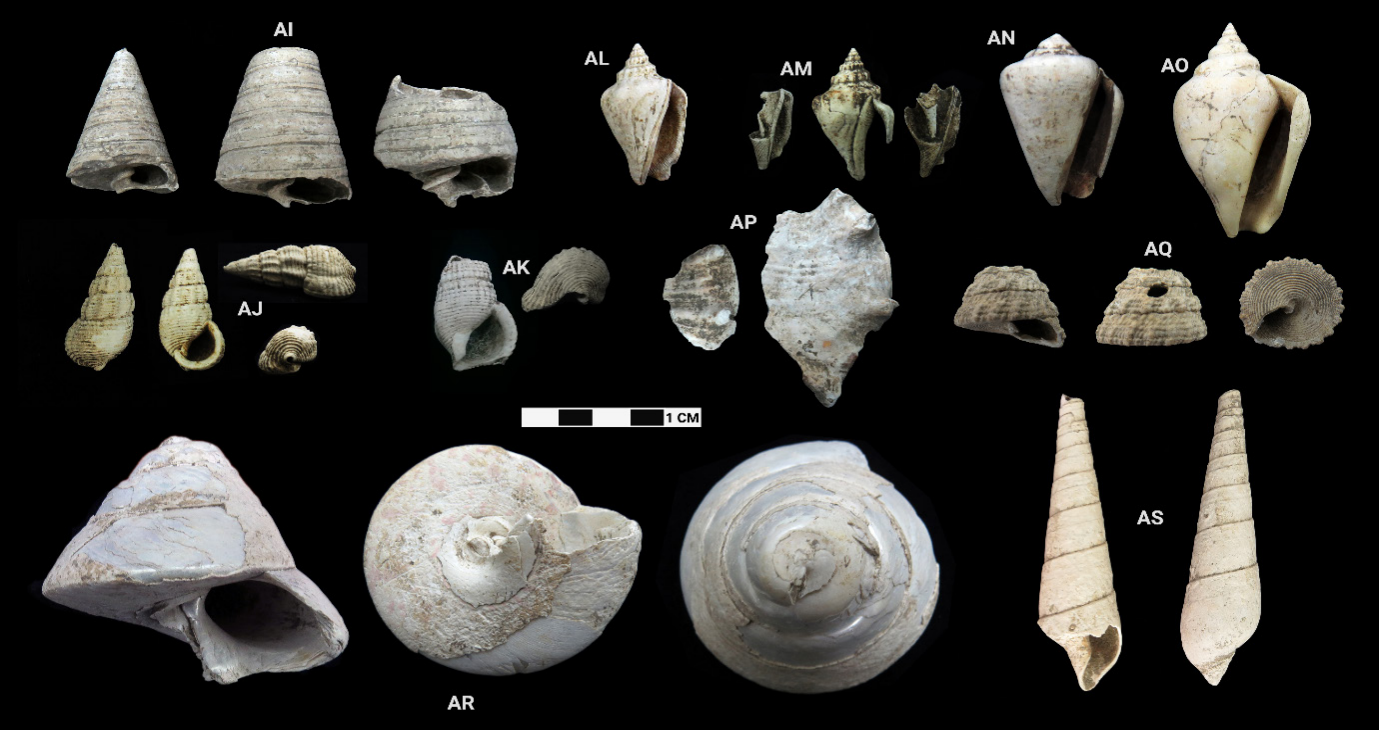
Gastropod specimens identified from archaeological sites in Semporna: (AI) *Telescopium telescopium* (MT/C/118), (AJ) *Terebralia sulcata* (MT/D/Q2/52), (AK) *Terebralia* sp. (BT02/P/Q2/15), (AL) *Canarium urceus* (BT08/C/11), (AM) *Canarium* sp. (BT08/C/10), (AN) *Conomurex luhuanus* (BT02/P/Q2/8), (AO) *Laevistrombus canarium* (MT/C/122), (AP) *Lambis* sp. (MT/D/Q1/204 & MT/D/Q2/170), (AQ) *Tectus fenestratus* (MT/D/Q2/49), (AR) *Rochia nilotica* (MT/D/Q1/94) and (AS) *Terebra* sp. (MT/D/Q2/99).

**Figure 9:**
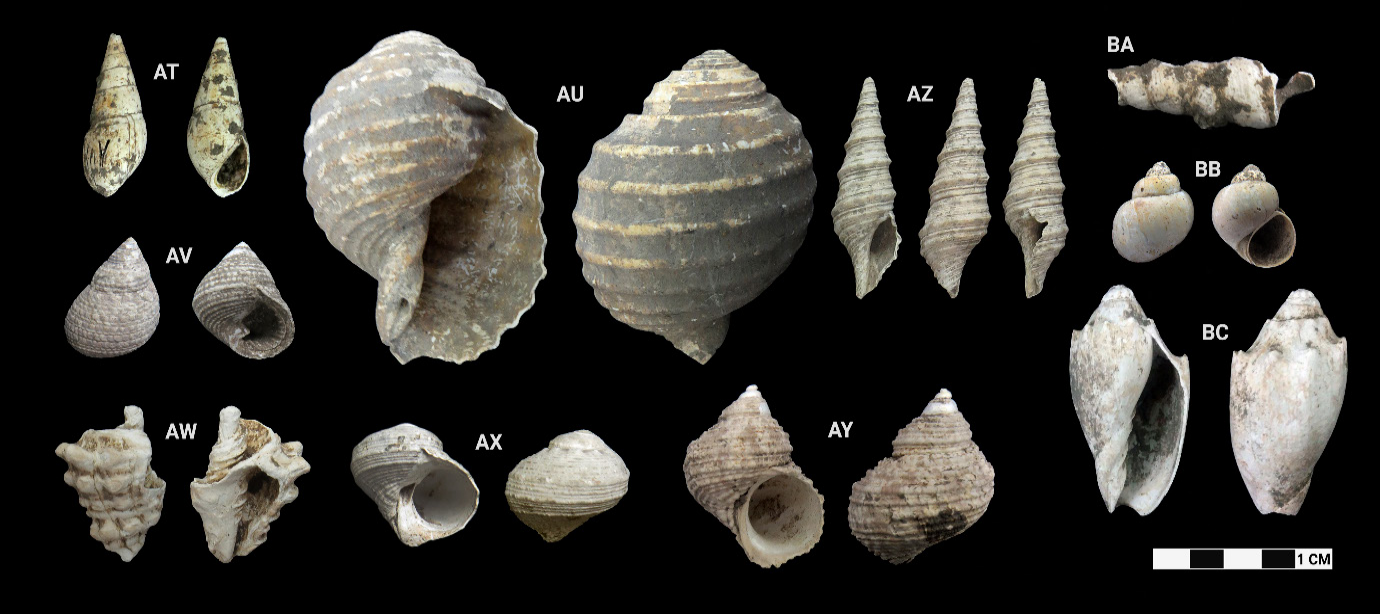
Gastropod specimens identified from archaeological sites in Semporna: (AT) *Stenomelania* spp. (BK/45), (AU) *Tonna* sp. (MT/C/114), (AV) *Monodonta labio* (MT/C/79), (AW) *Vasum turbinellus* (BT02/P/Q2/2), (AX) *Lunella cinerea* (MT/D/Q2/71), (AY) *Turbo bruneus* (MT/D/Q3/99), (AZ) *Unedogemmula indica* (MT/D/Q1/118), (BA) *Turritella* sp. (BK/46), (BB) *Filopaludina* cf. *javanica* (MT/C/77) and (BC) *Cymbiola vespertilio* (MT/D/Q1/128).

This research revealed a total of 90 taxa comprising 30 bivalve taxa and 60 gastropod taxa, of which 18 and 37 were identified to species level for each of the two classes, respectively. Tables 1 and 2 listed the identified bivalve and gastropod taxa, their habitat and morphological characteristics, respectively. Meanwhile, Figs. 2 to 9 illustrated all the identified bivalve and gastropod taxa respectively, along with the reference codes for the specimens depicted.

While it is easier for samples in complete state to be identified to species level, similar yet fragmented samples lacked the full characteristics for them to be identified to species level. Hence, these fragmented samples could only be identified to genus level. For example, fragmented samples similar to *Anadara antiquata* would only be identified as *Anadara* sp. instead (see Fig. 2 (A–B) and Table 1). Besides that, certain samples, despite being in complete state, were identified to genus level as it lacked diagnostic anatomic features for identification to species level, such as *Barbatia* sp. which could only be identified further to species by looking at the original colour of its outer surface (Poutiers 1998a). This was inevitable as our samples were dead specimens buried for thousands of years. Another example would be the gastropod specimen of *Sulcospira* spp., which could not be identified to the species level as it required observation on their embryonic shell and reproductive organs (Kohler & Glaubrecht 2006; Kohler & Dames 2009), which again was impossible for dead specimens. Hence, such specimens could only be identified as *Sulcospira* spp., with the possibility of it being *Sulcospira pageli*, *Sulcospira agrestis* or *Sulcospira infracostata* based on its shape and locality (Kohler & Dames 2009; Ng *et al*. 2017).

In archaeology, whenever a large number of mollusc remains is discovered, the basic assumption is that it is part of the food refuse (Waselkov 1987; Thomas 2015b). This assumption is often confirmed when the mollusc remains identified are edible species. In this study, most of the species of molluscs identified from the research sites were edible species, which made it clear that almost all of them had been exploited as food resources (Poutiers 1998a; 1998b). In fact, some of the species are still collected and consumed by the local Bajau Laut in Semporna today (Ridzwan 1993; Albert 2019). The Bajau Laut people live a semi-nomadic life, usually in boat-houses covered with thatches, although most had started to settle in houses on stilts along the coastline of Semporna but maintain a dominantly marine-oriented lifestyle working as fishermen and collecting marine products (Sather 1985; 2001; Halina 2007). Some of the Bajau Laut’s nomenclature of species identified in this study were ‘kohang’ (which includes various species of clams such as *Anadara antiquata*, *Tegillarca granosa* and *Gafrarium pectinatum*), ‘tehem’ (oysters under the family *Ostreidae*), ‘kima’ (giant clams under the subfamily Tridacninae), ‘mestuli’ (murex snails such as *Hexaplex chicoreum*), ‘sesoh’ (nerites under the genus *Nerita*), ‘binga-binga’ (turban snails such as *Turbo bruneus*), ‘baggungan’ (*Telescopium telescopium*), ‘limbi-limbi’ (*Terebralia sulcata*), and ‘buku-buku’ (*Pleuroploca trapezium*).

In addition, artificial man-made cuts found on certain species of the mollusc remains also indicated that they had been processed specifically for consumption. For instance, the long-spiralled gastropods such as *Sulcospira* spp., *Terebralia sulcata* and *Telescopium telescopium* had their apices removed. Such condition was a clear sign of food processing as it is well-known that apices of long-spiralled gastropods need to be cut so that their flesh can be easily sucked out from their apertures (Meehan 1982; Zuraina *et al*. 1994; Bujeng 2009; Nilus *et al*. 2010). On the other hand, small-sized gastropods such as *Nerita undata*, *Lunella cinerea*, *Paludomus everetti* and *Canarium urceus* were mostly found in their complete state because their meat could be easily extracted from their apertures using a sharp-pointed tool (Meehan 1982). Additionally, bivalves such as *Anadara antiquata*, *Tegillarca granosa*, and *Gafrarium pectinatum* were also found mostly in complete state because their meat could be easily extracted as their valves will open automatically when heated by boiling or roasting (Meehan 1982; Suniarti *et al*. 2019). These observations on the condition of specimens strengthen further the interpretation that the samples were food refuse.

The different habitats from which the identifiable molluscs originated from, as listed in Tables 1 and 2, suggested that subsistence activities took place in different environments, which required different gathering or foraging strategies and techniques. For instance, mollusc remains found on rocks in the intertidal zone such as *Nerita undata*, *Monodonta labio* and *Lunella cinerea* could be easily gathered during mid-tide (Poutiers 1998b). In addition, species living in seagrass beds such as *Anadara antiquata*, *Gafrarium pectinatum* and *Canarium urceus* could also be easily gathered during low tide by walking on the knee-deep shallow water. During high tide, certain tools might be needed such as a long pole with a net, locally known as ‘tangguk bergalah’ used by present day Malay fishermen to gather cockles from the sea bed while on a boat (Hamid 1989). Other species such as the giant clams (*Tridacninae* sp.) are often found attached on coral reefs and could be collected by diving into the ocean (Poutiers 1998a; Neo 2020). *Geloina expansa*, which is usually found at upper mangroves that are more freshwater than saltwater, is best collected during low tide because it burrows into the muddy bottoms (Meehan 1982; Albert & Bujeng 2021). In contrast, the mangrove or estuarine species *Terebralia sulcata* is usually collected during high tide because it will climb on stems and roots of mangrove trees (Poutiers 1998b).

Determining specific types of environment that the mollusc species originated from could only be done by identifying the specimens to their lowest taxonomic ranking as far as possible. In most cases, archaeological research often provided a general description of the original habitat of the mollusc remains, for instance by simply stating it as marine shells (see Datan 1993; Lertcharnrit 2005; Fajari & Kusmartono 2013; Sarjeant 2014). This study shows how important it is to do a detailed and comprehensive identification of the mollusc samples in order to be able to determine the types of environments the molluscs originated from. Some marine species of the same family can originate from different marine habitats, for example, *Hexaplex cichoreum* and *Chicoreus capucinus* are both from the Muricidae family but the former is found in rocky shores, while the latter is a coastal mangrove species (Poutiers 1998b; Tan & Oh 2002). Furthermore, the original habitat of the mollusc remains can provide useful insights into the past environment of the research sites. To date, most of the mollusc species identified in this study can still be found in Semporna today (Ridzwan 1993; Ridzwan & Kaswandi 1995). Therefore, it is highly likely that the past environments around the research sites from 3,000 to 800 years ago are closely similar to what it is today in Semporna, Sabah.

Next, for the species richness, despite the total number of taxa identified is 90 (30 for bivalves and 60 for gastropods), the unit of species richness, which is NTAXA, only calculates the number of taxa identified at the highest common taxonomic level. This is as seen in Tables 1 and 2, where the absolute frequency of NTAXA is denoted by f_i_, while the cumulative frequency of NTAXA is denoted by F_i_. Hence, the NTAXA value of bivalves is 21, while for gastropod is 46, which brought a total of NTAXA value for the whole assemblage from archaeological sites in Semporna to 67. Table 3 then compared the NTAXA values of bivalves and gastropods identified in the three archaeological sites, which showed that Melanta Tutup had the most species richness, followed by Bukit Tengkorak and Bukit Kamiri. This would give a possibility of a lower level of selectivity by the ancient community of Melanta Tutup when gathering molluscs compared with those from the other sites. This lower selectivity would mean that molluscs were gathered regardless of species without targeting any specific species. Perhaps the closer proximity of Melanta Tutup to the coastline (see Fig. 1) allowed the ancient community of the site to gather a wider array of molluscan species, hence not being selective, compared to the other sites. This is because marine species are generally considered more diverse compared to other environments (Rosenberg 2014).

However, it has also been widely documented that species richness is dependent on sample size. Smaller assemblages tend to have fewer taxa, and larger assemblages tend to have more taxa. In addition, a straightforward comparison of NTAXA between assemblages will inform more about differences in sampling rather than revealing the meaningful differences in richness ecologically, unless the sample sizes being compared are identical (Faith & Lyman 2019). Several statistical analyses can be used to evaluate species richness while taking sample size and sampling issues in consideration. The first one is sampling-to-redundancy, which aims to examine the adequacy of one’s sample by plotting the incremental addition of material in order to assess at what point a sample become representative of an assemblage’s population (Mitchell *et al*. 2016; Faulkner *et al*. 2018; Faith & Lyman 2019). The second one is rarefaction, which aims to estimate the expected NTAXA that would have been recovered had fewer specimens been collected (Woo *et al*. 2016; Faith & Lyman 2019). Next, regression analysis that uses one or more regression lines between sample size and NTAXA of multiple assemblages (Grayson & Delpech 1998; Faith & Lyman 2019). These statistical analyses would strengthen or correct interpretation made solely by comparison of NTAXA values, which is worth exploring further in future studies.

## SUMMARY AND CONCLUSION

This study had identified a total of 90 taxa of molluscs, with 55 identified to the species level, from the three archaeological sites in Semporna, Sabah. Some specimens could only be identified to genus, family or subfamily levels due to the lack of distinguishable elements because of their fragmented and discoloured state for being buried for thousands of years. The identified mollusc remains originated from a wide range of habitats, but marine species dominated, which suggested that the ancient community of Semporna during that time had a predominantly maritime-oriented economy, yet also exploited resources from freshwater and mangrove environments as well. Most of the mollusc remains were determined to be food leftovers because they were edible species based on data from malacological literature, evidence of food preparation found on specimens as well as local knowledge from the local Bajau Laut villagers in Semporna. By calculating the number of taxa at the highest common taxonomic level, the total NTAXA value from all three sites is 67, of which Melanta Tutup has the highest NTAXA value of 66, followed by Bukit Tengkorak (36) and Bukit Kamiri (17). This suggested a lower selectivity in gathering activities among ancient communities in Melanta Tutup, while those from Bukit Tengkorak and Bukit Kamiri probably had specific species targeted. However, such interpretation could be strengthened or altered by conducting statistical analyses that look into the relationship between NTAXA and sample size. Nevertheless, this study illustrated the importance of identification of mollusc remains uncovered from archaeological sites not only to interpret the behaviour of past humans, but also past environment and distribution of mollusc species in Semporna, Sabah.

## Figures and Tables

**Table 1:** The identified bivalves, NTAXA, and their habitats as well as morphological characteristics from the archaeological sites in Semporna, Sabah.

No.	Family	Subfamily/Genus/Species	Presence at site (+): Present, (−): Absent			NTAXA		Habitat	Characteristics

			BT	MT	BK	f_i_	F_i_		
1.	Arcidae	*Anadara antiquata*	+	+	+	1	1	Intertidal, seagrass bed with sandy or muddy substrate (Afiati 1999; Siahainenia *et al*. 2018)	Obliquely ovate and elongated to the posterior in outline, about 40 smooth radial ribs with a narrow median groove on top, inflated and forward umbo, numerous teeth on hinge plate
2.	Arcidae	*Anadara* sp.	−	+	−			Sandy or muddy intertidal to shallow subtidal sands (Poutiers 1998a)	Fragmented specimens elongated to the posterior in outline with smooth radial ribs and numerous teeth on hinge plate
3.	Arcidae	*Barbatia* sp.	−	+	−	1	2	Intertidal and shallow subtidal rocks or coral rubbles (Garcia & Oliver 2008)	Numerous teeth on hinge plate, exterior sculpted with smooth yet scaled-like radial ribs, narrow ligamental area that slants to the commissural plane of valves. Most specimens lack the exterior colour to be identified to species level
4.	Arcidae	*Tegillarca granosa*	+	+	+	1	3	Soft muddy bottoms of tidal flats in estuaries or bays (Joni *et al*. 2019; Yurimoto *et al*. 2014)	Ovate, commonly 6 cm in length, about 18 radial ribs bearing rectangular nodules that look like scales and numerous teeth on hinge plate
5.	Cardiidae	*Fragum unedo*	−	+	−	1	4	Clean sand in shallow water (intertidal to subtidal up to 50 m deep) (Poutiers 1998a)	Radial ribs on the exterior, stout hinge with anterior and posterior lateral and cardinal teeth on both valves, subquadrate in outline, bent posteriorly by a rounded radial angulation
6.	Cardiidae	*Vasticardium pectiniforme*	+	+	−	1	5	Coral or muddy sand flats in intertidal to shallow subtidal up to 20 m deep (Poutiers 1998a)	About 30 prominent, rounded and rugose radial ribs with transverse scales on sides and top on the anterior half of valve, stout hinge with anterior and posterior lateral and cardinal teeth on both valves, oblong-ovate in outline
7.	Cardiidae	*Tridacninae* sp.	+	+	−	1	6	Intertidal to shallow subtidal, usually around coral reefs (Poutiers 1998a)	Thick and heavy, presence of folds on the exterior. Mostly fragmented specimens
8.	Cardiidae	*Hippopus hippopus*	−	+	−			Sandy bottoms of coral reef, intertidal to shallow subtidal up to 6 m deep (Poutiers 1998a)	Thick and heavy, posteroventral margin bordered by interlocking crenulations, prominent ribbed radial folds with the shape of the dorsal free margin is triangulate
9.	Cardiidae	*Hippopus* sp.	+	+	+			Sandy bottoms of coral reef, intertidal to shallow subtidal (Poutiers 1998a)	Thick and heavy, posteroventral margin bordered by interlocking crenulations, rib-like folds. Mostly fragmented specimens
10.	Cardiidae	*Tridacna squamosa*	−	+	−			Attached to surfaces of coral reefs in intertidal and shallow subtidal up to 20 m deep (Poutiers 1998a)	Thick and heavy, presence of byssal gape at the posteroventral margin, prominent concentric lines and blade-like projections on its exterior
11.	Cardiidae	*Tridacna* sp.	+	+	−			Intertidal to shallow subtidal, usually around coral reefs (Poutiers 1998a)	Thick and heavy, presence of byssal gape at the posterovenral margin, prominent concentric lines. Mostly fragmented specimens
12.	Cyrenidae	*Batissa violacea*	−	+	−	1	7	Sandy or muddy beds of freshwater and brackish–water rivers and mangrove swamps (Mayor & Ancog 2016)	Round to ovate in outline, umbo points anteriorly and situates near midline of valves, presence of three diverging cardinal teeth and transversally striated lateral teeth on the hinge, concentrically striated
13.	Cyrenidae	*Geloina expansa*	+	+	−	1	8	Muddy and brackish, sometimes in almost freshwater areas of mangrove swamps (Yahya *et al*. 2018)	Trigonal-ovate in outline, prominent umbo points anteriorly and situates near midline of valves, presence of three diverging cardinal teeth and lateral teeth on posterior and anterior (1 each in left valve, 2 each in right valve), concentrically striated
14.	Cyrenidae	*Geloina* sp.	−	+	+			Generally mangrove areas (Hamli *et al*. 2015)	Prominent umbo points anteriorly and situates near midline of valves, presence of three diverging cardinal teeth and lateral teeth on posterior and anterior, concentrically striated. Mostly fragmented specimens
15.	Hemidonacidae	*Hemidonax donaciformis*	−	+	−	1	9	Clean coarse coral sand from low tide of intertidal to shallow subtidal (Poutiers 1998a)	Subtrigonal to wedge-shaped, transversely elongated in outline with anterior side equals or longer than the posterior side, smooth radial ribs, two unequal cardinal teeth and an elongated lateral tooth in each valve
16.	Lucinidae	*Austriella corrugata*	+	+	−	1	10	Deep burrower in mud of intertidal water of mangrove area (Glover *et al*. 2008)	Subcircular to quadrate in outline, rounded ventral margin, moderately inflated and sculpted with numerous prominent concentric growth lines, thin hinges with no teeth
17.	Lucinidae	*Austriella* sp.	−	−	+			Mangrove (Glover *et al*. 2008)	Mostly fragmented specimens that showed at least one characteristic of the *Austriella corrugata* above
18.	Mesodesmatidae	*Atactodea striata*	+	+	−	1	11	Sedentary filter–feeder found in sandy substrates of the intertidal zone (Ash *et al*. 2013)	Small, commonly 2.5 cm in length, poorly-defined umbo that curves toward posterior, relatively pronounced lateral teeth and outer surface sculpted with concentric ridges
19.	Ostreidae	*Saccostrea cuccullata*	+	+	−	1	12	Attached to hard substrates in marine, estuarine and mangrove areas, intertidal and shallow subtidal up to a depth of 5 m (Poutiers 1998a)	Solid, irregularly shaped, inequivalve where left valve is usually larger while the right valve is usually flat, presence of chomata (tiny folds, ridgelets-like in right valve with corresponding pits in left valve) all around the internal shell margin, with single adductor muscle scar and toothless hinge
20.	Ostreidae	*Ostreidae sp*.	−	+	+			Attached to substrates mainly in intertidal and shallow subtidal areas (Poutiers 1998a)	Fragmented specimens with irregular shapes, ligamental area with a shallow median groove and two lateral thickening and the presence of single adductor muscle scar
21.	Pectinidae	*Decatopecten* sp.	+	+	−	1	13	Sandy bottoms in intertidal and subtidal zones to a depth of 20 m (Poutiers 1998a)	Straight dorsal margin forming wing-like ears at both ends, single adductor muscle scar, radial sculpture on exterior with radial threads on ribs
22.	Spondylidae	*Spondylus* sp.	−	+	−	1	14	Mainly in relatively shallow waters cemented to hard substrate in coralline areas (Poutiers 1998a)	Stout shell with straight hinge line that has 2 strong teeth and 2 deep sockets, small ear on either side, single adductor muscle scar, exterior with irregular spinose radial ribs
23.	Pteriidae	*Isognomon isognomum*	−	+	−	1	15	Live in dense colonies attached to hard substrates in intertidal and shallow subtidal up to 20 m deep (Poutiers 1998a)	Presence of numerous transverse grooves on ligament area, strong posteroventral elongation and relatively long dorsal margin that expanded posteriorly, lustrous interior
24.	Pteriidae	*Isognomon* sp.	+	+	−				Presence of numerous transverse grooves on ligament area, lustrous interior, Mostly fragmented specimens
25.	Psammobiidae	*Asaphis violascens*	−	+	−	1	16	Sandy or gravel bottoms in intertidal and shallow subtidal up to 20 m deep (Poutiers 1998a)	Elongate-ovate in outline, shell inflated, slightly gaping posteriorly, exterior sculpted with strong, rounded and often forked radial ribs developed throughout the valves, 2 cardinal teeth in each valve
26.	Veneridae	*Anomalodiscus squamosus*	−	+	−	1	17	Fine sandy to muddy bottoms of intertidal and shallow subtidal, often near mangroves (Poutiers 1998a)	Subtrigonal in outline, commonly 3 cm in length, thick and prominent umbo, exterior sculpted with strong, dense radial riblets and low concentric cords, forming repeating squares-like sculpture
27.	Veneridae	*Gafrarium pectinatum*	+	+	−	1	18	Sandy bottoms of intertidal and shallow subtidal up to 20 m deep (Poutiers 1998a)	Thick, relatively compressed, exterior sculpted with strong nodulous radial ribs that diverge obliquely towards the anterior and posterior, strong hinge plate with 3 cardinal teeth and well developed anterior lateral teeth (1 in left valve, 2 in right valve)
28.	Veneridae	*Gafrarium divaricatum*	−	+	−	1	19	Clean, muddy sand or sandy gravel of intertidal and shallow subtidal up to 20 m deep (Poutiers 1998a)	Similar to *Gafrarium pectinatum*, but exterior is sculpted with fine concentric ridges, and fine radial ridges that diverge in the middle of the shell surface to form chevron lines
29.	Veneridae	*Periglypta puerpera*	+	+	−	1	20	Sandy or muddy bottom of intertidal and shallow subtidal up to 20 m deep (Poutiers 1998a)	Rounded-quadrate in outline with finely crenulated internal margin, exterior is sculpted with relatively fine concentric and radial ridges, strong hinge plate with three cardinal teeth in each valve, and a small, tubercle-shaped anterior lateral tooth in left valve
30.	Veneridae	*Pelecyora* sp.	−	+	−	1	21	Marine, sandy or muddy intertidal coast or mangrove (Moazzam & Moazzam 2020; Yahya *et al*. 2020; Al-Asif *et al*. 2021)	Ovate to bluntly trigonal in outline, sub-central umbo, large and superficial lunule, strong anterior lateral tooth on left valve opposite deep distant pit on right valve (Jukes-Browne, 1908)

*Note*: BT = Bukit Tengkorak; MT = Melanta Tutup; BK = Bukit Kamiri; f_i_ = absolute frequency; F_i_ = cumulative frequency

**Table 2:** The identified gastropods, NTAXA, and their habitats as well as morphological characteristics from the archaeological sites in Semporna, Sabah.

No.	Family	Subfamily/Genus/Species	Presence at site (+): Present, (−): Absent			NTAXA		Habitat	Characteristics

			BT	MT	BK	f_i_	F_i_		
1	Cerithiidae	*Clypeomorus batillariaeformis*	+	+	−	1	1	Sandy bottoms of reef flats and estuaries in the intertidal zone (Poutiers 1998b)	Small shell up to 3 cm in length, slightly inflated spire worlds, body whorl with 6 to 8 main spiral beaded cords and a strong, slightly oblique, axial varix on the left part of the dorsal side
2	Cerithiidae	*Rhinoclavis vertagus*	−	+	−	1	2	Sandy bottom in the intertidal and subtidal zones to a depth of about 13 m (Poutiers 1998b)	Only fragmented specimens were found. Distinguishable based on relatively low sculpture with fine spiral grooves and strong short axial folds under suture, becoming obsolete anteriorly and on body whorl
3	Conidae	*Conus* sp.	+	+	+	1	3	Clean or muddy-sand bottoms under rocks or corals in intertidal and shallow subtidal (Poutiers 1998b)	Cone-shaped, mostly fragmented specimens with a moderately low or flat spire and/or a well-developed body whorl tapering towards the narrow anterior end
4	Costellariidae	*Vexillum rugosum*	−	+	−	1	4	Sandy bottoms of the shallow subtidal waters, from low tide to a depth of about 10 m (Poutiers 1998b)	Strong axial ribs that formed prominent shoulder nodules on body whorls, elongated aperture that is anteriorly notched by a short siphonal canal, strong spiral folds on columella
5	Cyclophoridae	*Cyclophorus* sp.	−	+	−	1	5	Terrestrial (Egorov & Greke 2007)	Medium to large, smooth shell with fine spiral striation on body whorl, rather wide umbilicus
6	Cyclophoridae	*Opisthoporus* sp.	−	+	+	1	6	Terrestrial (Sang *et al*. 2020)	Shell discoidal in shape, moderately high spire, wide umbilicus, adorned with irregular axial brownish stripes
7	Cymatiidae	*Monoplex* sp.	−	+	−	1	7	Intertidal and subtidal sandy, rocky and reef environments (Poutiers 1998b)	Fragmented specimen, distinguished based on presence of axial varix, thickened and toothed outer lip, and moderately short siphonal canal that bent dorsalward
8	Cypraeidae	*Cypraea* sp.	+	+	−	1	8	Generally associated with coral reefs, but can be collected when empty shell washed ashore (Poutiers 1998b)	Ovate or oblong in shape, long and narrow aperture with raised transverse ridges or teeth on both lips. Most specimens found with fragmented dorsum
9	Ellobiidae	*Ellobium* sp.	+	+	−	1	9	Live amphibiously in nipa palm and mangrove swamps (Poutiers 1998b)	Fragmented specimens distinguished based on the ovate to cylindrical in shape with a rather short conical spire
10	Fasciolariidae	*Pleuroploca* sp.	+	+	−	1	10	Shallow water near rocky areas or inner reef flats to a depth of about 20 m (Snyder *et al*. 2012)	Fragmented specimens distinguished by its large shell, fusiform in shape with shoulder nodules, moderately long siphonal canal, roughly quadrate aperture that is finely pirate inside
11	Melongenidae	*Volema myristica*	−	+	−	1	11	Sandy mud flats of intertidal zone as well as brackish water and mangroves (Vermeij & Raven 2009)	Relatively small shell, biconical and quiet short in shape, spinose at the suture, short siphonal canal, sculpted with axial folds and rather thick spiral cords
12	Mitridae	*Nebularia eremitarum*	−	+	−	1	12	Abundant in the intertidal zone on coral reef environments (Poutiers 1998b)	Fusiform-ovate with a high tapering spire, rather narrow and elongated aperture that is anteriorly notched by a short siphonal canal, strong columellar folds
13	Muricidae	*Chicoreus brunneus*	−	+	−	1	13	Rocky, muddy and coral reef areas in the intertidal and subtidal zones (Poutiers 1998b)	High and acute spire, broad yet narrowly open anterior canal, crenulated outer lips without tooth-like process, three strong spinose axial varices with a single node between the varices
14	Muricidae	*Chicoreus capucinus*	+	+	−			Intertidal species common in mangroves (Tan & Oh 2002)	Similar to *Chicoreus brunneus*, but has three bluntly foliated axial varices on the body whorls with two nodes between the varices
15	Muricidae	*Chicoreus* sp.	+	+	+			Intertidal shore, estuary or mangrove (Poutiers 1998b)	Mostly fragmented specimens exhibiting spinose axial varices and narrowly open anterior canal
16	Muricidae	*Hexaplex cichoreum*	−	+	−	1	14	Rocky to muddy areas in the intertidal and subtidal zones (Poutiers 1998b)	Globose-ovate with a broad conical spire, wide body whorl, subcircular aperture, strongly spinose axial varices ranges from six to eight around its body whorl, moderately developed siphonal canal with spines
17	Muricidae	*Drupella margariticola*	−	+	−	1	15	Coral reef environment (Houart 2008; Claremont *et al*. 2011)	About 3 cm long, outer surface sculpted with axial nodose ribs with strong varices, high spire, ovate aperture with short siphonal canal
18	Muricidae	*Reishia bitubercularis*	−	+	−	1	16	Rocky intertidal areas (Tengku Hanidza *et al*. 2017)	Spirally ridged with axial stripes, spines on shoulder, short siphonal canal
19	Nassariidae	*Nassarius arcularia*	−	+	−	1	17	Sand bottoms in coral reef areas in intertidal and subtidal zones (Poutiers 1998b)	Small yet stout shell, large columellar callus that forms a smooth flat shield over the ventral side of shell, spire whorls with thick axial ribs, aperture lirate inside
20	Nassariidae	*Nassarius coronatus*	−	+	−			Intertidal and subtidal sand (Poutiers 1998b)	Squat and thick shell with a fairly high spire, large columellar callus that forms a smooth flat shield over the ventral side of shell, spire whorls with shoulder nodules
21	Nassariidae	*Nassarius dorsatus*	+	+	−			Intertidal scavengers, common at the mid-tide level on sheltered mud-flats (McKillup & McKillup 1997)	Specimens found with broken apices, smooth and glossy body whorl with only spiral grooves at the anterior end, outer lip has varixed backside, narrow columellar callus, aperture lirate inside
22	Nassariidae	*Nassarius* sp.	−	+	−			Intertidal and subtidal sands or reef areas (Poutiers 1998b)	Fragmented specimens exhibiting aperture that is lirate inside, bear calloused columellar, fairly high conical spire
23	Naticidae	*Polinices* sp.	−	+	−	1	18	On sandy bottoms, often associated with coral reefs in the intertidal and subtidal zones (Poutiers 1998b)	Pear-shaped shell, umbilicus filled by callus, short spire, smooth and glossy exterior, large semicircular aperture
24	Neritidae	*Nerita albicilla*	−	+	−	1	19	Rocky shores, in the upper mid-tidal pools on damp and submerged rocks (Poutiers 1998b)	Thick, globose shell with flat spire, wide and flat columellar shield, numerous small denticles at inner margin of outer lips, numerous distinct pustules over the surface of columellar shield
25	Neritidae	*Nerita histrio*	−	+	−			Tree trunks and stilt roots in mangrove or breakwater rocks in muddy sand flats (Tan & Clements 2008)	Globose shell sculpted with rough and unevenly raised spiral cords with prominent axial sculpturing, few obsolete teeth at center of columellar edge, low to nearly flat spire
26	Neritidae	*Nerita plicata*	−	+	−			Holes and crevices in rocks of supralittoral zone (Tan & Clements 2008)	Thick, turbinate shell with moderately high conical spire, numerous coarse rounded spiral ribs on the body whorl, a calloused and convex columellar shield with elongated and square teeth on inner margin
27	Neritidae	*Nerita undata*	+	+	−			In between rocks in the upper intertidal shores, but usually only appear at dusk (Tan & Clements 2008)	Spirally sculptured exterior with a moderately high spire, inner margin of columellar shield contains 3 to 5 teeth with the uppermost tooth shaped like a square, dentate outer lip with a distinctly larger tooth at the upper end
28	Neritidae	*Nerita* sp.	+	+	+			Generally rocky intertidal areas (Poutiers 1998b)	Any fragmented specimens that resemble the spire, columellar shield and outer lip of nerites
29	Neritidae	*Neritina cf. pulligera*	−	+	−	1	20	Fast-flowing freshwater streams and rivers or brackish waters in close proximity to ocean (Gerlach *et al*. 2016	Flat spire lower than the outer lip, flat parietal wall that is larger than the aperture, axial growth lines on exterior
30	Neritidae	*Vittina turrita*	+	+	+	1	21	Common in brackish water of mangrove and estuaries, occasionally in freshwater streams (Poutiers 1998b)	Thin yet solid shell that is elongated-ovate in outline due to having an elevated and conical spire that is often eroded at the apex, smooth outer surface, columellar shield and outer lip. Most specimens still retain their colour: black with white axial stripes
31	Olividae	*Oliva* sp.	−	+	−	1	22	In sand flats of intertidal and shallow subtidal zones (Poutiers 1998b)	Thick, porcelaneous shell that is elongate-ovate, short spire, elongated aperture with a wide and short siphonal canal, inner lip calloused with columella obliquely grooved
32	Ovulidae	*Ovula ovum*	+	+	−	1	23	Common on the coral *Sarcophyton* to a depth of 20 m (Poutiers 1998b)	Easily distinguished by the globular egg-shape of shell that is smooth and porcelaneous, short yet stout anterior and posterior extremities, long and narrow aperture with thickened outer lip that is irregularly dentate
33	Pachychilidae	*Sulcospira* spp.	+	+	+	1	24	In relatively fast running freshwater such as rivers, streams or creeks (Marwoto & Isnaningsih 2012)	Conical to turreted-shaped in outline, smooth sculpture with occasional presence of axial ribs, spiral ridges and/or spines, rounded or pointed basal lips with spiral striae at the bottom of the final body whorl. Most specimens have broken apices
34	Paludomidae	*Paludomus everetti*	+	+	+	1	25	Forest streams and in the vicinity of limestone caves (Ng *et al*. 2017)	Oblong-globe shape, distinctive spiral impressed lines on the succeeding whorls below the suture, aperture is ovate or less acuminate or tapering to a point, thick inner lip, spire shorter than aperture
35	Paludomidae	*Paludomus* sp.	−	+	−			Freshwater rivers or streams, especially rocky-bottomed, narrow, shallow, shady and slow-moving (Amarasinghe & Krishnarajah 2009)	Any fragmented specimens that exhibited at least one characteristic of *Paludomus everetti* above
36	Planaxidae	*Planaxis sulcatus*	−	+	−	1	26	In intertidal rocky shores, around the mid to low tidal zones during low tide (Tan & Low 2014)	Solid and moderately elongated, up to 3.5 cm in shell height, inflated whorls each sculpted with 5 or 6 incised spiral lines, deeply impressed suture, ovate aperture that is denticulate within
37	Potamididae	*Cerithidea* cf. *quoyii*	−	+	−	1	27	On mangrove trees in marine intertidal water and occasionally brackish area (Reid 2014)	Small shell with axial and spiral sculpture, periphery of the last whorl is angular. All specimens are fragmented, with no aperture and outer lip, hence could be compared to other species
38	Potamididae	*Telescopium telescopium*	+	+	−	1	28	Soft mangrove muds (Zaman & Jahan 2013)	High-conical shell with many spire whorls sculpted with three large and one narrow spiral cords alternately with deep grooves, relatively small and obliquely quadrangular aperture, twisted columella with a central spiral ridge with a short siphonal canal. Mostly fragmented specimens
39	Potamididae	*Terebralia sulcata*	+	+	−	1	29	In mangrove areas, and occasionally brackish estuaries (Poutiers 1998b; Putri & Patria 2018)	Elongated shell sculpted with deeply incised suture that has four or five spiral cords alternate in each suture along with numerous axial ridges resembling a pattern of square nodules, thick outer lip that is widely flared and expanded anteriorly that connects to the base of columella forming a short tube-like bottom that looks like an umbilicus
40	Potamididae	*Terebralia* sp.	+	+	+			Mangrove	Any fragmented specimens that exhibited at least one characteristic of *Terebralia sulcata* above
41	Strombidae	*Canarium urceus*	+	+	−	1	30	Sandy mud bottoms in intertidal and subtidal up to 40 m deep (Poutiers 1998b)	Elongated-ovate in shape, high spire, a more drawn out siphonal canal, a smooth central portion of columella that is pirate (having fine thread-like lines) at both ends, body whorl sculpted with fine spiral grooves near anterior end and outer lip margin only
42	Strombidae	*Canarium* sp.	+	+	+			Intertidal	Any fragmented specimens that exhibited at least one characteristic of *Canarium urceus* above
43	Strombidae	*Conomurex luhuanus*	+	+	−	1	31	Sandy bottoms of coral reefs, lagoons, seagrass and coral rubbles in intertidal and shallow subtidal up to 20 m deep (Poutiers 1998b)	Small, short spire with a long and narrow aperture, somewhat similar to cone shells but has a well-developed stromboid notch of the outer lip, black or chocolate brown inner lip on the aperture
44	Strombidae	*Laevistrombus canarium*	+	+	−	1	32	Muddy sand and algae bottoms in intertidal and subtidal up to 55 m deept (Poutiers 1998b)	Solid, heavy, globose and smooth shell, with moderately high conical spire, long aperture and a greatly expanded-thickened-rounded outer lip with its upper end projecting slightly upward with a stromboid notch
45	Strombidae	*Lambis* sp.	+	+	−	1	33	Usually associated with coral reefs and lagoons, either in intertidal or subtidal (Poutiers 1998b)	Mostly fragmented specimens distinguished based on the features of the flaring outer lips with marginal digitations
46	Tegulidae	*Tectus fenestratus*	+	+	−	1	34	Common on rocky shores in the muddy areas of intertidal zone (Naung 2018; Poutiers 1998b)	Conical shell with a flat base, squarish aperture, columella with a concave spiral fold, and sculpted with thick, rounded oblique ribs on the body whorl. Mostly found with broken apices
47	Tegulidae	*Tectus* sp.	−	+	−			Rocky shores	Any fragmented specimens with a flat base and squarish aperture
48	Tegulidae	*Rochia nilotica*	−	+	−	1	35	Common in coral reefs, especially shallow, high-energy portions of barrier and fringing reefs (Poutiers 1998b)	Large, thick and heavy shell, conical in shape, nearly smooth body whorl, nearly flat base
49	Terebridae	*Terebra* sp.	−	+	−	1	36	Sandy intertidal and subtidal (Poutiers 1998b)	Elongated and sharply conical shell with a high, many-whorled spire and relatively small quadrae to triangular aperture. All fragmented specimens with broken apices and aperture
50	Thiaridae	*Stenomelania* spp.	+	+	+	1	37	Freshwater, or near brackish water of estuaries (Apiraksena *et al*. 2020)	Elongated and pointed shell, relatively large body whorl with straight profile of the spire whorls
51	Tonnidae	*Tonna* sp.	−	+	−	1	38	Mainly living on sandy bottoms where seagrasses abounded in the subtidal zone (Poutiers 1998b)	Thin, globose yet large shell with a short spire but large body whorl, exterior sculpted spirally with relatively flat ribs or cords
52	Trochidae	*Monodonta labio*	−	+	−	1	39	Common on rocky shores in intertidal zone, grazing on microalgae (Takada 2001; Poutiers 1998b)	Small yet thick shell, with an asymmetrical cone with spirals of rounded bumps, a convex base and a single large tooth-shaped structure on the columella at the aperture
53	Turbinellidae	*Vasum turbinellus*	+	−	−	1	40	Common in rocky bottoms on reef flats in the intertidal and subtidal zones (Poutiers 1998b)	Only fragmented specimens found, but could easily be distinguished based on biconical shape, the blunt spines on body, and strong unequal columellar folds
54	Turbinidae	*Lunella cinerea*	−	+	−	1	41	Common among rocks or gravel in rocky intertidal shore (Poutiers 1998b)	Shell looks more like a dome than turbinated, commonly 3 cm in length, body whorl sculpted with fine nodulose spiral cords, low spire, wide open umbilicus
55	Turbinidae	*Turbo bruneus*	−	+	−	1	42	Common in rocky shores and coral reef areas in shallow subtidal zone (Poutiers 1998b)	Turbinate in shape, sculpted with strong spiral cords and very fine axial ridges, large and oval aperture that extends to half of the total length of shell and flares a little at the anterior end of the smooth columella
56	Turbinidae	*Turbo* sp.	+	+	−			Mainly living in intertidal and shallow subtidal zones on rocky and coral reef habitats (Poutiers 1998b)	Any fragmented specimen that has at least one of the characteristics of *Turbo bruneus* above
57	Turridae	*Unedogemmula indica*	−	+	−	1	43	Common on muddy bottoms in shallow subtidal to a depth of about 50 m (Poutiers 1998b)	Fusiform shell strongly keeled at the shoulder, angulated sides of the whorls, bear posterior notch of outer lip, outer surface with spiral cords
58	Turritellidae	*Turritella* sp.	−	+	+	1	44	Shallow subtidal up to 30 m deep (Poutiers 1998b)	Mostly fragmented specimens, distinguished based on the numerous whorls sculpted with spiral cords
59	Viviparidae	*Filopaludina* cf. *javanica*	−	+	−	1	45	Freshwater: rivers, lakes, ponds, swamps (Marwoto & Nurinsiyah 2009)	Globose shell, fine axial growth lines, round aperture, pointed yet blunt spire
60	Volutidae	*Cymbiola vespertilio*	+	+	−	1	46	On muddy sand or mud bottom in intertidal and subtidal zone to a depth of 20 m (Poutiers 1998b)	Elongate-ovate in shape, short and conical spire, spiny tubercles on shoulder of body whorl, bear oblique columella folds, wide and long aperture about 80% the total length of shell

*Note*: BT = Bukit Tengkorak; MT = Melanta Tutup; BK = Bukit Kamiri; f_i_ = absolute frequency; F_i_ = cumulative frequency

**Table 3:** NTAXA values of the three archaeological sites from Semporna, Sabah.

Site/Class	Bukit Tengkorak	Melanta Tutup	Bukit Kamiri
Bivalves	12	21	6
Gastropods	23	45	11

Total	36	66	17
